# pyrpipe: a Python package for RNA-Seq workflows

**DOI:** 10.1093/nargab/lqab049

**Published:** 2021-06-01

**Authors:** Urminder Singh, Jing Li, Arun Seetharam, Eve Syrkin Wurtele

**Affiliations:** Bioinformatics and Computational Biology Program, Iowa State University, Ames, IA 50014, USA; Center for Metabolic Biology, Iowa State University, Ames, IA 50014, USA; Department of Genetics Development and Cell Biology, Iowa State University, Ames, IA 50014, USA; Center for Metabolic Biology, Iowa State University, Ames, IA 50014, USA; Department of Genetics Development and Cell Biology, Iowa State University, Ames, IA 50014, USA; Genome Informatics Facility, Iowa State University, Ames, IA 50014, USA; Bioinformatics and Computational Biology Program, Iowa State University, Ames, IA 50014, USA; Center for Metabolic Biology, Iowa State University, Ames, IA 50014, USA; Department of Genetics Development and Cell Biology, Iowa State University, Ames, IA 50014, USA

## Abstract

The availability of terabytes of RNA-Seq data and continuous emergence of new analysis tools, enable unprecedented biological insight. There is a pressing requirement for a framework that allows for fast, efficient, manageable, and reproducible RNA-Seq analysis. We have developed a Python package, (pyrpipe), that enables straightforward development of flexible, reproducible and easy-to-debug computational pipelines purely in Python, in an object-oriented manner. pyrpipe provides access to popular RNA-Seq tools, within Python, via high-level APIs. Pipelines can be customized by integrating new Python code, third-party programs, or Python libraries. Users can create checkpoints in the pipeline or integrate pyrpipe into a workflow management system, thus allowing execution on multiple computing environments, and enabling efficient resource management. pyrpipe produces detailed analysis, and benchmark reports which can be shared or included in publications. pyrpipe is implemented in Python and is compatible with Python versions 3.6 and higher. To illustrate the rich functionality of pyrpipe, we provide case studies using RNA-Seq data from GTEx, SARS-CoV-2-infected human cells, and *Zea mays*. All source code is freely available at https://github.com/urmi-21/pyrpipe; the package can be installed from the source, from PyPI (https://pypi.org/project/pyrpipe), or from bioconda (https://anaconda.org/bioconda/pyrpipe). Documentation is available at (http://pyrpipe.rtfd.io).

## INTRODUCTION

Since its inception, RNA-Seq has become the most widely used method to quantify transcript levels (1,2). A researcher can leverage the now-massive RNA-Seq data in public databases, encompassing samples from multiple species, organs, genotypes and conditions (3). Integrated reanalysis of aggregations of these diverse RNA-Seq samples enables exploration of changes in gene expression over time and across different biological conditions (4).

A major challenge in analysis of RNA-Seq datasets is implementing data processing pipelines in an efficient, modular, and reproducible manner (5–8). Most bioinformatics tools are executable programs, executed via a command-line interface (CLI), that must be specified inside a scripting language for automated execution. Thus, writing bioinformatics pipelines as Perl, Bash or Python scripts is a common practice among bioinformaticians. Scripting is powerful and flexible. However, plain scripting has several significant downsides (9). First, especially for complicated pipelines, bash scripts can be difficult to develop or maintain. Second, for beginners it is hard to write bash scripts in a robust manner that can handle exceptions or resolve errors dynamically. Writing multiple commands along with all the parameters in a single bash script often becomes hard to read, understand, and modify. Third, bash scripts do not provide an easy-to-use framework for building modular pipelines. Fourth reproducibility of methods is best-practice in computing, being required by more and more journals (10), and scripting alone has significant limitations for reproducible bioinformatics (9). Fifth, scripts often contain significant ‘boilerplate code’ as the user repeats commands and parameters in the script. This results in challenges, particularly for complex pipelines. Managing the tool’s parameters and making changes becomes difficult and error prone. Controlling parameters is essential for reproduciblity but are difficult to document, and hard to track if not well documented. Moreover, no straightforward framework exists to define tools and parameters and modify them at runtime.

Here we present Python RNA-Seq pipeliner, pyrpipe, a lightweight Python package for users to code and execute computational pipelines in an object-oriented manner, in pure Python. No new *workflow* syntax that is specific to pyrpipe is required. pyrpipe delivers an intuitive framework to easily *import* any Linux/macOS executable command or third-party tool as reusable Python objects.

**Figure 1.**
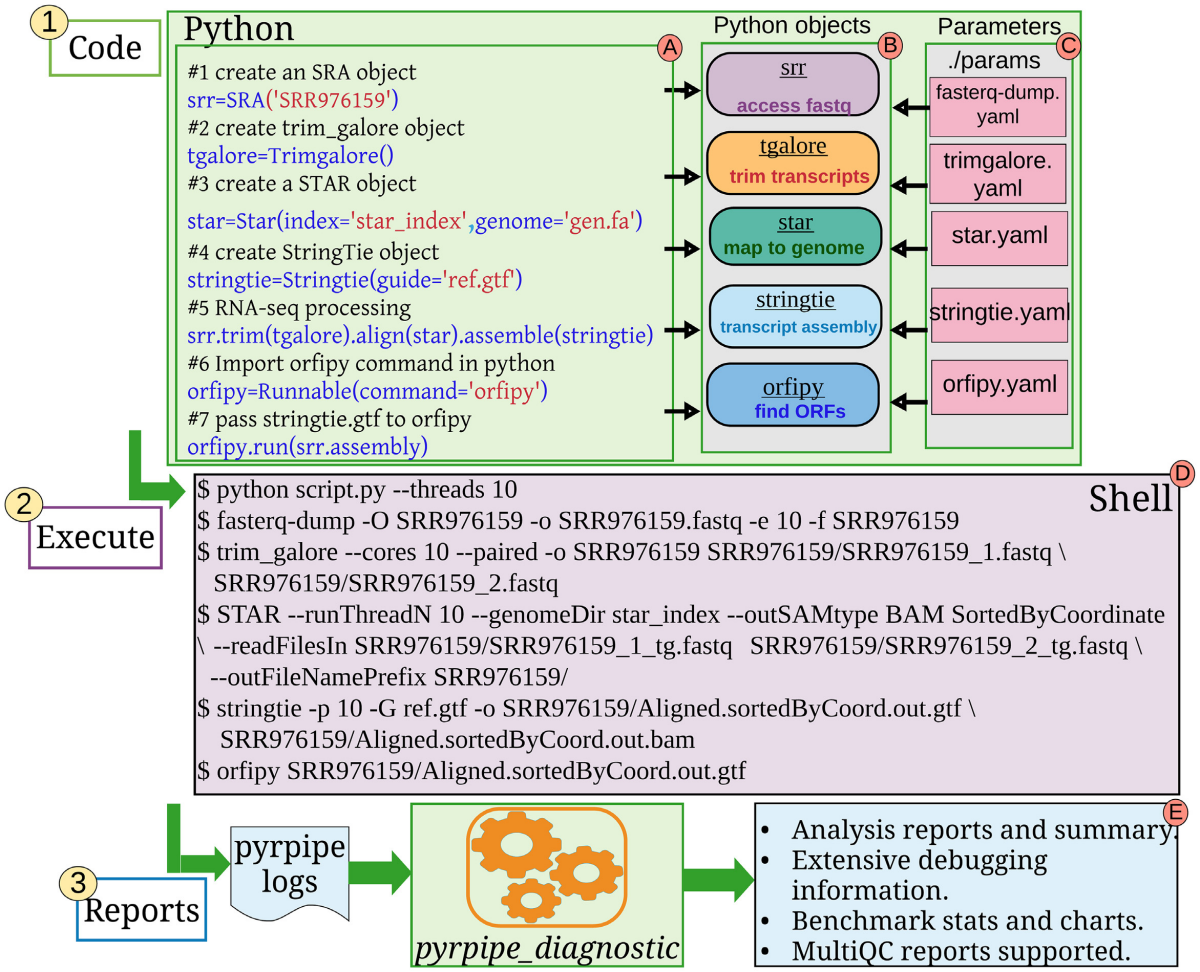
The pyrpipe framework. This very simple example illustrates the relationship between the Python code that the user writes for pyrpipe, the corresponding Python objects, the YAML parameter files, the corresponding shell script, and the output. The user need only define the NCBI-SRA Run Accessions and the tools to be used, the rest is automatic. Here, a single RNA-Seq run is specified; alternatively, thousands of runs could be processed. A key advantage of pyrpipe is that it can be used to easily create complex workflows that are intuitive, understandable, reproducible, and modifiable. pyrpipe can automatically load and resolve tool parameters from YAML files; this allows the user to facilely modify and document parameters (an example of a YAML file is in Supplementary Figure S7). pyrpipe is represented by the green boxes. The user writes the code in Python (blue text), creating Python *objects* of specific pyrpipe classes that provide APIs to RNA-Seq tools. To execute the full pipeline, the user need to run only the Python file, e.g. “python script.py –threads 10″, to designate executing the pipeline using 10 threads (Box A). Each *object* encapsulates specific *methods* and *data* (Box B). For example, each *SRA* object stores the directory path for the associated raw RNA-Seq data that is used as the default directory by pyrpipe to output files from different RNA-Seq processing steps, i.e., trimming, alignment, assembly or quantification. Tool parameters, if supplied in YAML files, are automatically loaded and stored in the corresponding pyrpipe object (Box C). During processing, shell commands are automatically constructed and executed by the pyrpipe APIs; pyrpipe provides this comprehensive output of bash commands so that the user can easily monitor the status of the job. (Box D). After execution, the *pyrpipe_diagnostic* tool generates extensive data analyses and diagnostic reports from the logs. These enable users to summarize, share, benchmark or debug their pipelines (Box E).

Using the pyrpipe framework, users can implement RNA-Seq downloading and processing pipelines in a single go, quickly and intuitively. In addition, we have designed APIs to popular RNA-Seq tools and incorporated these into pyrpipe to enable coherent RNA-seq processing – from managing the raw data, to trimming, alignment, and assembly or quantification. pyrpipe’s simple API design allows for automated access to publicly available NCBI-SRA RNA-Seq data (11) allowing users to quickly implement pipelines for harmonized re-analysis of these datasets (Figure 1).

pyrpipe will be helpful for users looking for a robust approach to write pipelines in pure Python. Compared to plain Bash, Perl, or Python scripting, pyrpipe provides many helpful features for building reproducible and easy-to-share pipelines. These features include: extensive logging and reports; loading tool options from YAML files to easily modify and document tool parameters; a dry-run mode to check dependencies and targets before implementing large-scale analysis; resuming of jobs if they are interrupted; and saving pyrpipe sessions.

pyrpipe can be used for quick prototyping of RNA-Seq processing pipelines because of the ease in swapping out pyrpipe objects, such as substituting Stringtie (12) for Cufflinks (13) for transcript assembly. pyrpipe pipelines can be easily scaled using a workflow manager, including the popular Snakemake (7), NextFlow (8) or Toil (14). The workflow management system then can scale and manage jobs on clusters and schedule independent jobs for parallel processing, facilitating scalable pipelines and optimizing resource usage. Meanwhile, pyrpipe, whether used independently or as part of a workflow management system, facilitates ease-of-implementation, reproducibility, understandability, and modification of the RNA-Seq processing pipeline.

## MATERIALS AND METHODS

### Overview

We developed pyrpipe to provide a light-weight Python framework for implementing bioinformatics or other computational analysis pipelines. The pyrpipe framework include: (i) high-level APIs to popular RNA-Seq tools; (ii) a general API to import any executable command/tool into Python, enabling use of any bioinformatics tool and (iii) extensive monitoring and logging details of the commands that are executed. Thus, pyrpipe allows users to *import* any Linux/macOS executable command/tool into the Python ecosystem and implement pipelines in pure-Python incorporating their own Python code, existing Python libraries and third-party programs. To execute the commands, pyrpipe uses Python’s *subprocess* library but adds many useful features and options. The commands executed via pyrpipe are automatically logged, monitored, and can be flexibly controlled using pyrpipe options. pyrpipe is packaged as a Python library and can be installed via PyPI or conda. An advantage of using the Python platform is that it is widely used, free, flexible, object-oriented, has high-level data structures (15–18), and a growing repository of >200 000 packages and tools.

### The pyrpipe framework

pyrpipe enables users to code pipelines in an object-oriented manner, using specialized API ‘classes’ provided by pyrpipe. Each class in pyrpipe is designed to work with a particular processing tool, for example, the *Star* class implements the necessary functionality to use the STAR tool (19) for RNA-Seq alignment via pyrpipe. Users can create specific *objects* of these classes and use the *objects* in their Python scripts (Figure 1). Each RNA-Seq processing tool is fully accessible via these *objects* and the user is not required to remember the full usage syntax of that tool, hence promoting *abstraction*. Instead, the data and parameters required by these tools are *encapsulated* within the respective *objects*. For example, when creating a *Star* object, its index and other parameters are saved with the object (Figure 1). pyrpipe provides flexible parameter management (Figure 1). If no parameters are provided by the user, the tool is executed with its default parameters. We recommend that users fully understand and use the best parameters for their pipelines.

Tools performing similar types of RNA-Seq processing steps are grouped together in a single pyrpipe module, and are designed to have identical APIs. This enables their *objects* to be easily interchangeable in pipelines, promoting reusability and modification. For example, the classes ‘Star’ and ‘Hisat2’, both in the pyrpipe ‘mapping’ module, implement the *build_index* and *perform_alignment* functions. Thus, changing a *Star* object with a *Hisat2* object is straightforward. See Supplementary Data for implementation details.

### APIs for RNA-Seq processing

pyrpipe provides high-level APIs, to access full functionality of 11 popular RNA-Seq analysis tools that expedite and enhance implementation of RNA-Seq pipelines that can be readily shared, modified, or reused, including a dedicated module to facilitate access and management of the extensive RNA-Seq data available from the National Center for Biotechnology Information Research Sequence Read Archives (NCBI-SRA) database (3).

These API classes are implemented inside several highly cohesive modules: (*sra*, *mapping*, *alignment*, *quant*, *qc*, *tools*). Each module has been designed to capture steps integral to RNA-Seq analysis: (i) access NCBI-SRA and manage raw RNA-Seq data; (ii) quality control; (iii) read alignment; (iv) transcript assembly and (v) transcript quantification. (Supplementary Table S1 and Supplementary Figures S1 and S2). We have built and integrated these APIs into the pyrpipe package, such that any RNA-Seq processing pipeline can be intuitively executed by the researcher while writing minimal code (Figure 1).

By default, all output files are consistently named and managed by pyrpipe, and put in the same directory as the RNA-Seq data files. Users can provide a different output directory.

### Flexibility in pipeline execution, debugging, and pipeline sharing

pyrpipe flexibility extends to enabling the user to choose how to execute and handle exceptions and errors to modify their pipeline’s behavior.

Users can create checkpoints in the pipeline, save the current pyrpipe*session*, and resume later. This is particularly useful for running different blocks of a workflow in different environments that can optimize resource usage. For example, on a typical high performance computing (HPC) cluster, a researcher might use a dedicated data-transfer node to retrieve data from SRA and then use compute nodes for data processing.

pyrpipe allow users to *dry run* the pipeline, during which commands are printed to screen, but not executed; thus, any potential error in the pipeline can be detected and fixed before using it to process large amounts of data. In addition, pyrpipe can skip execution of commands for which the output files are already present, saving computer time. Users can deploy the *–force* option to re-execute these commands (See Supplementary Data).

pyrpipe’s logging features enable efficient error detection and reports (Figure 1). Errors and extensive environment information, such as operating system and Python version, along with version and path information for each program used within the pipeline, are all logged. pyrpipe logs are saved in JavaScript Object Notation (JSON) format for parsing by pyrpipe and other software (Supplementary Table S2).

The *pyrpipe_diagnostic* command can be invoked to generate comprehensive reports about the analysis, benchmark comparisons (Supplementary Figure S3), shell scripts and MultiQC reports (20). These reports, along with the Python scripts, can be shared or included with publications to ensure reproducibility.

The default pyrpipe behaviour for logging, dry-run, and reports, can be modified by supplying pyrpipe with specific options via command-line or by specifying these in a *pyrpipe_conf.yaml* file.

### Reproducible analysis

Reproducibility can be a major challenge in bioinformatics studies because of heavy computational intensive tasks that depend on a number of software and system libraries. Reproducibility can be ensured by controlling execution environments via environment managers such as Anaconda, container systems such a Docker, or isolated virtual machines (5).

pyrpipe is a Python package available through bioconda (21) and can be installed and managed within conda environments, containers or VMs. We have included in pyrpipe documentation the recommended way of installing the required tools, with version information (Supplementary Table S1), for RNA-Seq analysis via bioconda (21).

Besides the user controlling the execution environment, pyrpipe adds several layers to enhance reproducibility of analysis. pyrpipe creates a local copy of the pipeline script so that user has access to the exact pipeline code later. pyrpipe logs the MD5 checksums of the pipeline script and any input files provided as arguments. Thus, the user can verify which scripts and input files were used in the analysis. We recommend users to use a version control software such as Git to keep a track of the changes to the scripts.

pyrpipe allows and encourages users to define separate YAML files for the tool parameters. This enables the user to modify, manage, share and reproduce computational analysis on different data and platforms. Further, pyrpipe logs contain detailed information about all the tools/commands used and their versions, which can be utilized to re-build the environments.

## RESULTS

We evaluated pyrpipe by three case studies, each illustrating a different aspect of what the tool can accomplish and how new functionality can be added.

### Case study 1: Scaling up pyrpipe to process 17 328 RNA-Seq samples from non-diseased human tissues

This case study demonstrates the ability of pyrpipe to process large amounts of data—17 328 human RNA-Seq samples from the Genotype-Tissue Expression (GTEx V8) (22). We developed and implemented our pipeline to identify expressed human orphan genes, as well as annotated genes, in diverse tissues using pyrpipe. This pipeline cohesively automated the steps of RNA-Seq processing into a single. It : (i) downloaded data from 17 328 raw GTEx RNA-Seq samples via AnVil (anvilproject.org); (ii) aligned the reads of each sample to the human reference genome using STAR (19); (iii) assembled transcripts using Stringtie (12); (iv) merged transcriptomes from individual samples into a consistent assembly using orfipy (23), Mikado (24) and Taco (25) and (v) quantified the annotated and unannotated transcripts using Salmon (26) (see Supplementary Figure S4 for details). This pipeline was run on the PSC Bridges HPC system (https://www.psc.edu/resources/bridges/). The pipeline was scaled to run multiple batches of RNA-Seq samples in parallel on multiple nodes. Code and data for this project is available https://github.com/urmi-21/pyrpipe/tree/master/case_studies/GTEx_processing.

**Figure 2.**
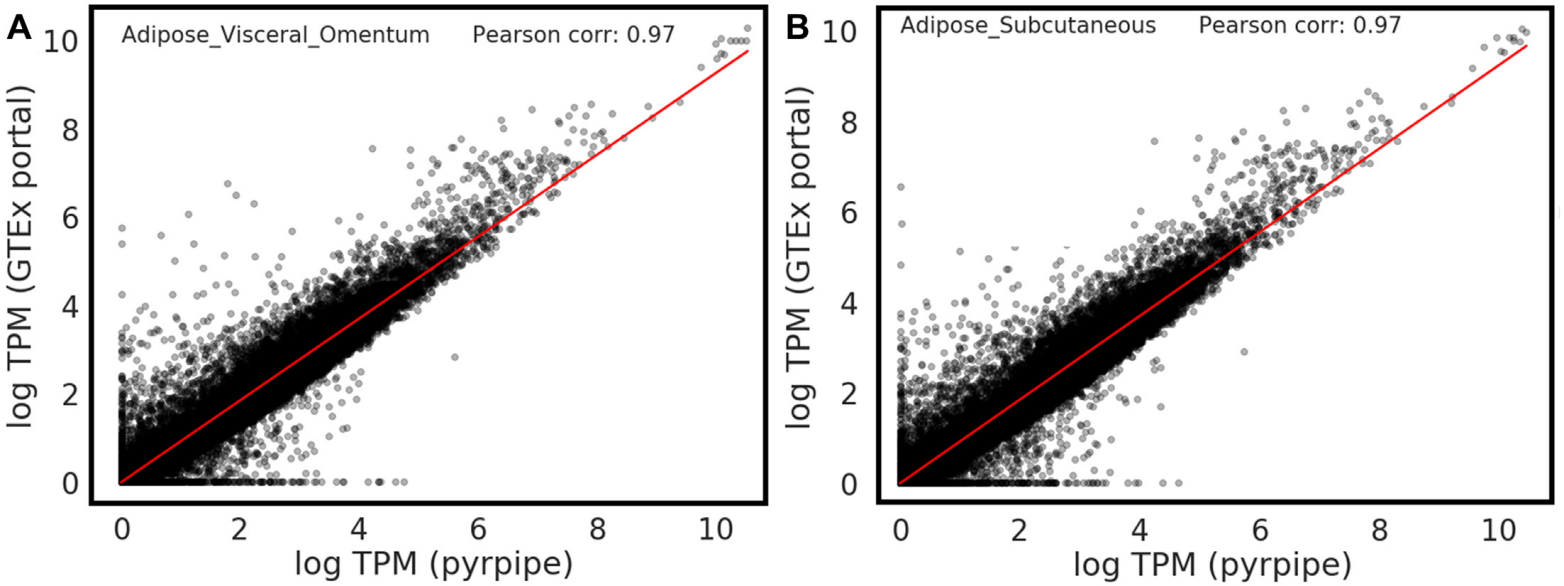
Comparison of median TPMs for two tissue types. (**A**) Visceral Adipose and (**B**) subcutaneous Adipose. Y-axis shows the logged median TPMs computed by the GTEx portal pipeline. X-axis shows the logged median TPMs computed by our pipeline implemented with pyrpipe. Pearson correlations are 0.97%. Differences in quantification of several 100 genes are likely due differences in reference annotations. Code and data to reproduce this plot and, to compare other tissue types are available at https://github.com/urmi-21/pyrpipe/tree/master/case_studies/GTEx_processing.

To assess the results of our pipeline, we have compared the expression of annotated genes identified by the pyrpipe pipeline with those reported in GTEx (a pipeline that only quantifies the annotated genes). This comparison showed good accordance between expression values from the two pipelines. We compare the median TPMs of annotated genes for two types of adipose tissue, as processed by pyrpipe and by the GTEx portal (Figure 2).

### Case study 2: Integrating pyrpipe within a workflow manager to quantify gene expression in COVID-19 samples for exploratory analysis

We implemented pyrpipe within two workflow management systems, Snakemake (7) and NextFlow (8), selecting these specifically because they are widely used by the bioinformatics community (27). Snakemake and NextFlow were independently used to implement, manage and execute the pipeline for multiple RNA-Seq samples in parallel on a single cluster.

We used this pipeline to quantify RNA-Seq data from a COVID-19 study of circulating monocytes (28), and provide output that can be directly analyzed by biologists, using the versatile Java software for exploratory analysis of large datasets, MetaOmGraph (MOG).

Specifically, we used pyrpipe to seamlessly download 29 RNA-Seq samples from NCBI-SRA (accession SRP287810) and quantify expression of annotated transcripts using Salmon’s selective alignment approach (26,29). This study analyzed RNA-Seq data from circulating monocytes derived from individuals with COVID-19 and healthy individuals, treated and untreated with hydroxychloroquine. The final transcript and gene level TPMs from each sample are merged into a single file to create a MetaOmGraph (4) project (*MOGproject-monocytes-60241genes-29samples-HCQtreat-2021-1-17*) for exploratory data analysis.

Using MetaOmGraph for rapid exploration of the data, we identified genes that show differential expression patterns between COVID-19-diseased individuals (*n* = 20) versus healthy individuals (*n* = 9).

**Figure 3.**
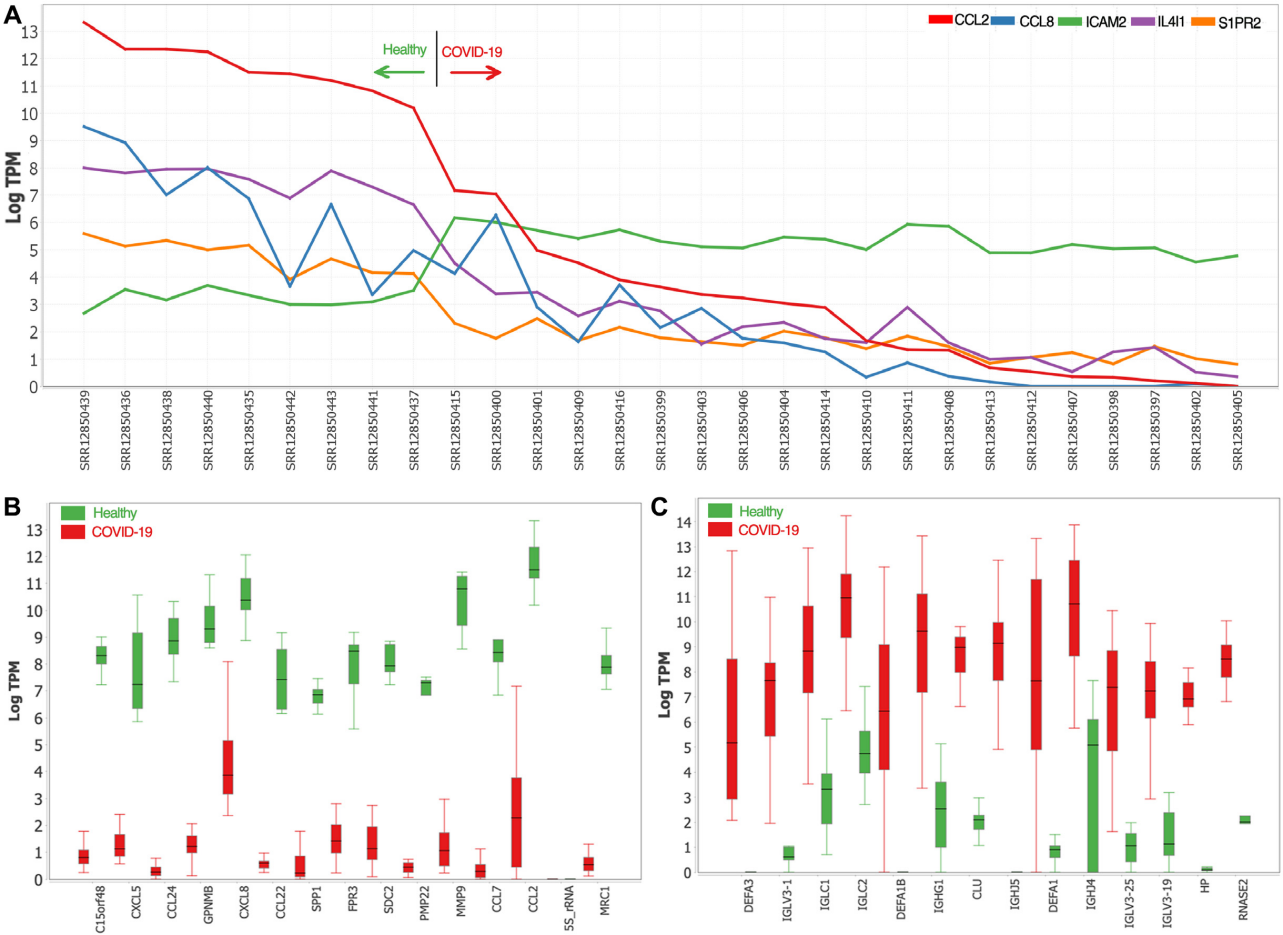
Exploratory analyses and visualization using MetaOmGraph (4) of RNA-Seq data dervived from monocytes of COVID-19 diseased and healthy individuals. The data were downloaded from NCBI-SRA and processed using pyrpipe integrated into the workflow manager, Snakemake. Raw reads from 29 samples of monocytes derived from individuals with COVID-19 (*n* = 20) and healthy (*n* = 9) individuals (SRP287810) were downloaded and processed. Over 60,000 genes are represented in each sample of processed data. For quick preliminary exploration of the data, using MetaOmGraph, we identified genes with more than two-fold change in TPM values with Benjamini-Hochberg adjusted p-value <0.002 for the non-parametric Mann–Whitney test. (**A**) Line chart showing expression pattern of genes non-linearly associated with CCL2 (estimated via Mutual Information (40)) in COVID-19 and healthy individuals. (**B**) Fourteen genes with highest fold change in healthy versus COVID-19 diseased individuals. (**C**) Fourteen genes with highest fold change in COVID-19 diseased versus healthy individuals.

Nine of the fourteen genes most highly overexpressed in monocytes from healthy individuals are involved in the biological process, neutrophil chemotaxis (GO:0030593; Bonferoni corrected *P*-value of 5.229E–10); these include six CCL- and CXL-type chemokines. Interestingly, in lung tissues, CCL2 and other chemokine expression are decreased by ACE2, but up-regulated during a COVID-19-induced cytokine storm (30). Of the 14 genes highly expressed in monocytes from COVID-19-diseased individuals but not in healthy indivduals (Figure 3C), 12 participate in immune effector (GO:0002252; Bonferoni corrected *P*-value of 3.161E–12), including nine defensins or immunoglobins. These biological processes are also associated strongly with COVID-19 in neutrophils (31). Functional designations were obtained using ToppGene (https://toppgene.cchmc.org) (Supplementary File 1).

The code, data, and MetaOmGraph project are available at https://github.com/urmi-21/pyrpipe/tree/master/case_studies/Covid_RNA-Seq.

### Case study 3: Use of pyrpipe for *de novo* transcriptome assembly

We used a new, high-quality genome of *Zea mays* B73 cultivar (https://doi.org/10.1101/2021.01.14.426684) as reference genome, and gathered RNA-Seq data from ten diverse samples (B73 cultivar), representing different tissue and development stages, for *de novo* transcriptome assembly (Supplementary Figure S5). Our pipeline identified a total of 57 916 distinct transcripts. Of these, 38,881 transcripts were homologous to UniProt proteins (32,33). These transcripts could be non-coding RNAs (ncRNAs), low-level ‘noise’ (34), or pseudogenes; others might represent as yet unannotated genes encoding conserved proteins. The remaining 6,306 transcripts, with no similarity to any protein in the database, could be ncRNAs, ‘noise’; others are likely to be as yet unannotated species-specific (‘orphan’) genes (35). The transcript length and GC content distribution for transcripts with conserved CDS and transcripts are shown in Supplementary Figure S6. The mean length of non-homologous transcripts (1290 nt) is shorter than conserved transcripts (1981 nt); mean GC content is indistinguishable (50.8% versus 50.6% ). The median expression of non-homologous transcripts across the 10 RNA-Seq samples analyzed is lower than the median expression of conserved transcripts; however, in each sample, hundreds of non-homologous transcripts are more highly expressed than the mean of the conserved genes. These characteristics follow the same trend as those of the conserved and orphan genes in the well-characterized *Arabidopsis thaliana* genome (36). Pipeline scripts, downstream analysis code and data are available at https://github.com/lijing28101/maize_pyrpipe.

### Comparison of pyrpipe to existing Python libraries that can be used for RNA-Seq analysis

Several Python libraries enable workflows to be specified. However, they do not provide a dedicated API suite for RNA-Seq data analysis. Instead, these frameworks depend on the user to explicitly write the commands and provide data.

We compared pyrpipe with two such Python libraries that allow specifying bioinformatics pipeline - Ruffus (37) and Pypiper (http://code.databio.org/pypiper/). Ruffus is a Python library for specifying and executing workflows. Ruffus allows users to specify pipeline tasks using several *‘decorator’* functions. Pypiper is a Python package for coding pipelines in Python. It provides the ‘PipelineManager’ class which a user can employ to execute commands in a serial manner. Pypiper has a built-in toolkit, NGSTk, to allow users to generate commonly used bioinformatics shell commands. These functions return commands as *string* objects that can be passed to ‘PipelineManager’ for execution. Table 1 compares pyrpipe features with Ruffus and Pypiper.

**Table 1.** Comparison of pyrpipe features with Ruffus and Pypiper. *For parallel execution support, pyrpipe easily can be integrated with a workflow management system, e.g. case study 2

Feature	pyrpipe	Ruffus	Pypiper
Latest version	0.0.5	2.8.4	0.12.1
Latest update	2021	2020	2019
API to RNA-Seq tools	✓	✗	✗
Import tools as objects	✓	✗	✗
Auto-load tool parameters	✓	✗	✗
Dry run mode	✓	✗	✗
Resume Interrupted	✓	✓	✓
Exception handling	✓	✓	✓
Parallel execution support*	✗	✓	✗
Logs/reports	✓/✓	✓/✗	✓/✓

## DISCUSSION AND CONCLUSION

The pyrpipe package allows users to code and implement RNA-Seq workflows in an object-oriented manner, purely using Python. pyrpipe is intended for any user who analyzes RNA-Seq data- beginner or advanced. APIs to RNA-Seq tools make it straightforward to code RNA-Seq processing pipelines. Access to NCBI-SRA is automated, such that users can readily retrieve raw read RNA-Seq data. The downloaded raw RNA-Seq data and data files are automatically managed, and consistently accessed through *SRA* objects. Users need not keep track of data files or paths, as these are integrated with pyrpipe objects. pyrpipe enhances the re-usability of the code-blocks, cutting down development time for new pipelines from existing code base. It also improves re-usability of the workflows, because all the parameters that needs to be adjusted for new analyses could be read from YAML files. pyrpipe workflows can be modified using Python’s control flow abilities and a user can create complex, reproducible, workflow structures. Any third party tool, executable command, or script can be integrated into pyrpipe for additional data processing capability. pyrpipe logs and reports enable debugging and reproducibility.

Analysis of the 17 328 GTEx RNA-Seq samples was easily scaled using pyrpipe alone, by creating smaller *batches* of samples and submitting the processing jobs in parallel, on an HPC system with a slurm job scheduler.

When building more complex and scalable workflows, it may be more efficient to integrate pyrpipe into a workflow management system. This can easily be done, as shown in our second case study. Workflow management systems are developed for robust implementation of computational pipelines; nevertheless, they differ significantly in terms of workflows, definitions, job scheduling, and features (6–8,14). For example, Snakemake uses a ‘pull-based’ strategy to check for specific output files and schedule jobs accordingly (6,7), whereas Nextflow uses a ‘push-based’ scheme in which a ‘process’ defined in the workflow pushes its outputs to downstream ‘processes’ (8,38). The SciPipe (6) workflow library is written in the GO language; similar to Nextflow it implements dataflow based task scheduling. Toil (14) provides explicit application programming interfaces (APIs) for defining static or dynamic tasks and supports common workflow language (CWL) and multiple cloud environments (9,14). Hence, users need to make informed decisions if choosing a workflow management system (27) for pyrpipe.

The modular design of pyrpipe permits users to write *pythonic* code, which is designed to read, manage, and share. Because of the rapid emergence of new bioinformatics tools, this design feature is particularly important. From a developer’s perspective, pyrpipe’s modularity facilitates reuse and extensibility; new tools/APIs can be easily integrated into pyrpipe and promotes sustainability.

Compared to plain Bash or Python scripting, pyrpipe provides many handy features for writing reproducible, robust and flexible pipelines in pure Python. Being simple, powerful, and easy to learn, Python has become one of the most popular languages among biologists and bioinformaticians; furthermore, a wide variety of tools are available in Python (15,16). Keeping this in mind, we designed a general framework such that pyrpipe is fully extendable to include any third-party tool, while writing minimal code. This design lets Python users integrate their own customized APIs into the pyrpipe ecosystem and thus incorporate diverse functionality into their pipelines. We have provided an example in the documentation (https://pyrpipe.readthedocs.io/en/latest/tutorial/api.html).

pyrpipe will appeal to users who are looking for a simple way to deploy small or large scale RNA-Seq processing pipelines, and to make these pipelines accessible to the community. pyrpipe supports the model of reproducible open science. Straightforward and seamless integration, execution, and sharing of RNA-Seq workflows make it an ideal choice for users with less computational expertise, as well as seasoned bioinformaticians. Writing Python code using pyrpipe is intuitive and maintainable. Leveraging Python language’s flow control and exception-handling abilities, users can quickly create complex and dynamic pipelines. Moreover, downstream analysis and data manipulation steps can be directly integrated into pyrpipe pipelines via Python.

## DATA AVAILABILITY

We subscribe to FAIR data and software practices (39). pyrpipe source code is available at https://github.com/urmi-21/pyrpipe. pyrpipe source code (v0.0.5) can be accessed via DOI: 10.5281/zenodo.4448373. The pyrpipe package can be installed from the source, from PyPi (https://pypi.org/project/pyrpipe) or from bioconda (https://anaconda.org/bioconda/pyrpipe). Extensive documentation to guide users on how to use pyrpipe and the APIs implemented within it is available on Read the Docs (http://pyrpipe.rtfd.io). We encourage contributions from the bioinformatics community (Contribution guide along with a Code of Conduct to guide new contributors is available at https://github.com/urmi-21/pyrpipe We hope to see pyrpipe evolve as a community driven project.

## Supplementary Material

lqab049_Supplemental_Files

## References

[1] Mortazavi A. , Williams B.A., McCue K., Schaeffer L., Wold B. Mapping and quantifying mammalian transcriptomes by RNA-Seq. Nat. Methods. 2008; 5:621–628. 18516045 10.1038/nmeth.1226 PMC13303166

[2] Stark R. , Grzelak M., Hadfield J. RNA sequencing: the teenage years. Nat. Rev. Genet. 2019; 20:631–656. 31341269 10.1038/s41576-019-0150-2

[3] Kodama Y. , Shumway M., Leinonen R. The Sequence Read Archive: explosive growth of sequencing data. Nucleic Acids Res. 2011; 40:D54–D56. 22009675 10.1093/nar/gkr854 PMC3245110

[4] Singh U. , Hur M., Dorman K., Wurtele E.S. MetaOmGraph: a workbench for interactive exploratory data analysis of large expression datasets. Nucleic Acids Res. 2020; 48:e23. 31956905 10.1093/nar/gkz1209 PMC7039010

[5] Grüning B. , Chilton J., Köster J., Dale R., Soranzo N., van den Beek M., Goecks J., Backofen R., Nekrutenko A. et al. Practical computational reproducibility in the life sciences. Cell syst. 2018; 6:631–635. 29953862 10.1016/j.cels.2018.03.014 PMC6263957

[6] Lampa S. , Dahlö M., Alvarsson J., Spjuth O. SciPipe: a workflow library for agile development of complex and dynamic bioinformatics pipelines. GigaScience. 2019; 8:giz044. 31029061 10.1093/gigascience/giz044 PMC6486472

[7] Köster J. , Rahmann S. Snakemake—a scalable bioinformatics workflow engine. Bioinformatics. 2012; 28:2520–2522. 22908215 10.1093/bioinformatics/bts480

[8] Di Tommaso P. , Chatzou M., Floden E.W., Barja P.P., Palumbo E., Notredame C. Nextflow enables reproducible computational workflows. Nat. Biotechnol. 2017; 35:316. 28398311 10.1038/nbt.3820

[9] Leipzig J. A review of bioinformatic pipeline frameworks. Brief. Bioinformatics. 2017; 18:530–536. 27013646 10.1093/bib/bbw020 PMC5429012

[10] Wittenburg P. Open science and data science. Data Intell. 2021; 3:95–105.

[11] Sherry S. , Xiao C. Ncbi sra toolkit technology for next generation sequence data. Plant and Animal Genome XX Conference (January 14-18, 2012). 2012; Plant and Animal Genome.

[12] Pertea M. , Pertea G.M., Antonescu C.M., Chang T.-C., Mendell J.T., Salzberg S.L. StringTie enables improved reconstruction of a transcriptome from RNA-seq reads. Nat. Biotechnol. 2015; 33:290. 25690850 10.1038/nbt.3122 PMC4643835

[13] Trapnell C. , Williams B.A., Pertea G., Mortazavi A., Kwan G., Van Baren M.J., Salzberg S.L., Wold B.J., Pachter L. Transcript assembly and quantification by RNA-Seq reveals unannotated transcripts and isoform switching during cell differentiation. Nat. Biotechnol. 2010; 28:511. 20436464 10.1038/nbt.1621 PMC3146043

[14] Vivian J. , Rao A.A., Nothaft F.A., Ketchum C., Armstrong J., Novak A., Pfeil J., Narkizian J., Deran A.D., Musselman-Brown A. et al. Toil enables reproducible, open source, big biomedical data analyses. Nat. Biotechnol. 2017; 35:314. 28398314 10.1038/nbt.3772 PMC5546205

[15] Suarez C.G.H. , Burbano M.E.G., Guerrero V.A.B., Tovar P.A.M. Bioinformatics software for genomic: a systematic review on GitHub. 2018; PeerJ doi:19 November 2018, preprint: not peer reviewed 10.7287/peerj.preprints.27352v3.

[16] Mariano D. , Ferreira M., Sousa B.L., Santos L.H., de Melo-Minardi R.C. A brief history of bioinformatics told by data visualization. Brazilian Symposium on Bioinformatics. 2020; Springer 235–246.

[17] Kossaifi J. , Panagakis Y., Anandkumar A., Pantic M. Tensorly: tensor learning in python. J. Mach. Learn. Res. 2019; 20:925–930.

[18] Kanterakis A. , Iatraki G., Pityanou K., Koumakis L., Kanakaris N., Karacapilidis N., Potamias G. Towards reproducible bioinformatics: the OpenBio-C scientific workflow environment. 2019 IEEE 19th International Conference on Bioinformatics and Bioengineering (BIBE). 2019; IEEE Computer Society 221–226.

[19] Dobin A. , Davis C.A., Schlesinger F., Drenkow J., Zaleski C., Jha S., Batut P., Chaisson M., Gingeras T.R. STAR: ultrafast universal RNA-seq aligner. Bioinformatics. 2013; 29:15–21. 23104886 10.1093/bioinformatics/bts635 PMC3530905

[20] Ewels P. , Magnusson M., Lundin S., Käller M. MultiQC: summarize analysis results for multiple tools and samples in a single report. Bioinformatics. 2016; 32:3047–3048. 27312411 10.1093/bioinformatics/btw354 PMC5039924

[21] Grüning B. , Dale R., Sjödin A., Chapman B.A., Rowe J., Tomkins-Tinch C.H., Valieris R., Köster J. Bioconda: sustainable and comprehensive software distribution for the life sciences. Nat. Methods. 2018; 15:475–476. 29967506 10.1038/s41592-018-0046-7 PMC11070151

[22] Aguet F. , Guigó Serra R., Montgomery S.B. et al. Genetic effects on gene expression across human tissues. Nature. 2017; 550:204–213. 29022597 10.1038/nature24277 PMC5776756

[23] Singh U. , Wurtele E.S. orfipy: a fast and flexible tool for extracting ORFs. Bioinformatics. 2021; btab090. 33576786 10.1093/bioinformatics/btab090 PMC8479652

[24] Venturini L. , Caim S., Kaithakottil G.G., Mapleson D.L., Swarbreck D. Leveraging multiple transcriptome assembly methods for improved gene structure annotation. GigaScience. 2018; 7:giy093. 10.1093/gigascience/giy093 PMC6105091 30052957

[25] Niknafs Y.S. , Pandian B., Iyer H.K., Chinnaiyan A.M., Iyer M.K. TACO produces robust multisample transcriptome assemblies from RNA-seq. Nat. Methods. 2017; 14:68–70. 27869815 10.1038/nmeth.4078 PMC5199618

[26] Patro R. , Duggal G., Love M.I., Irizarry R.A., Kingsford C. Salmon provides fast and bias-aware quantification of transcript expression. Nat. Methods. 2017; 14:417. 28263959 10.1038/nmeth.4197 PMC5600148

[27] Jackson M. , Kavoussanakis K., Wallace E.W. Using prototyping to choose a bioinformatics workflow management system. PLoS Comput. Biol. 2021; 17:e1008622. 33630841 10.1371/journal.pcbi.1008622 PMC7906312

[28] Rother N. , Yanginlar C., Lindeboom R.G., Bekkering S., van Leent M.M., Buijsers B., Jonkman I., de Graaf M., Baltissen M., Lamers L.A. et al. Hydroxychloroquine Inhibits the trained innate immune response to interferons. Cell Rep. Med. 2020; 100146. 33377122 10.1016/j.xcrm.2020.100146 PMC7762774

[29] Srivastava A. , Malik L., Sarkar H., Zakeri M., Almodaresi F., Soneson C., Love M.I., Kingsford C., Patro R. Alignment and mapping methodology influence transcript abundance estimation. Genome Biol. 2020; 21:1–29. 10.1186/s13059-020-02151-8 PMC7487471 32894187

[30] Merad M. , Martin J.C. Pathological inflammation in patients with COVID-19: a key role for monocytes and macrophages. Nat. Rev. Immunol. 2020; 20:355–362. 32376901 10.1038/s41577-020-0331-4 PMC7201395

[31] Aschenbrenner A.C. , Mouktaroudi M., Kraemer B., Oestreich M., Antonakos N., Nuesch-Germano M., Gkizeli K., Bonaguro L., Reusch N., Baßler K. et al. Disease severity-specific neutrophil signatures in blood transcriptomes stratify COVID-19 patients. Genome Med. 2021; 13:1–25. 33441124 10.1186/s13073-020-00823-5 PMC7805430

[32] Bateman A. , Martin M.-J., Orchard S., Magrane M., Agivetova R., Ahmad S., Alpi E., Bowler-Barnett E.H., Britto R., Bursteinas B. et al. UniProt: the universal protein knowledgebase in 2021. Nucleic Acids Res. 2020; 49:D480–D489. 10.1093/nar/gkaa1100 PMC7778908 33237286

[33] Altschul S.F. , Gish W., Miller W., Myers E.W., Lipman D.J. Basic local alignment search tool. J. Mol. Biol. 1990; 215:403–410. 2231712 10.1016/S0022-2836(05)80360-2

[34] Pertea M. , Shumate A., Pertea G., Varabyou A., Breitwieser F.P., Chang Y.-C., Madugundu A.K., Pandey A., Salzberg S.L. CHESS: a new human gene catalog curated from thousands of large-scale RNA sequencing experiments reveals extensive transcriptional noise. Genome Biol. 2018; 19:1–14. 30486838 10.1186/s13059-018-1590-2 PMC6260756

[35] Singh U. , Wurtele E.S. Genetic novelty: how new genes are born. Elife. 2020; 9:e55136. 32072921 10.7554/eLife.55136 PMC7030788

[36] Arendsee Z.W. , Li L., Wurtele E.S. Coming of age: orphan genes in plants. Trends Plant Sci. 2014; 19:698–708. 25151064 10.1016/j.tplants.2014.07.003

[37] Goodstadt L. Ruffus: a lightweight Python library for computational pipelines. Bioinformatics. 2010; 26:2778–2779. 20847218 10.1093/bioinformatics/btq524

[38] Strozzi F. , Janssen R., Wurmus R., Crusoe M.R., Githinji G., Di Tommaso P., Belhachemi D., Möller S., Smant G., de Ligt J. et al. Scalable workflows and reproducible data analysis for genomics. Evolutionary Genomics. 2019; Springer 723–745. 10.1007/978-1-4939-9074-0_24 PMC7613310 31278683

[39] Wilkinson M.D. , Dumontier M., Aalbersberg I.J., Appleton G., Axton M., Baak A., Blomberg N., Boiten J.-W., da Silva Santos L.B., Bourne P.E. et al. The FAIR Guiding Principles for scientific data management and stewardship. Scientific Data. 2016; 3:1–9. 10.1038/sdata.2016.18 PMC4792175 26978244

[40] Daub C.O. , Steuer R., Selbig J., Kloska S. Estimating mutual information using B-spline functions–an improved similarity measure for analysing gene expression data. BMC Bioinformatics. 2004; 5:118. 15339346 10.1186/1471-2105-5-118 PMC516800

