# Supplementary material for: pyrpipe: a Python package for RNA-Seq workflows
Source: NAR Genom Bioinform. 2021 Jun 1;3(2):lqab049. doi: 10.1093/nargab/lqab049 (PMC8168212; doi:10.1093/nargab/lqab049)
Supplement: lqab049_Supplemental_Files [file lqab049_supplemental_files.zip › pyrpipe_NARGB_SI.pdf]

`pyrpipe`: a python package for RNA-Seq workflows

Urminder Singh[1,2,3], **Jing Li**[2,3], **Arun Seetharam**[4], **and Eve Syrkin Wurtele**[1,2,3*]

[1]Bioinformatics and Computational Biology Program, Iowa State University, Ames, IA 50014 USA
[2]Center for Metabolic Biology, Iowa State University, Ames, IA 50014, USA
[3]Department of Genetics Development and Cell Biology, Iowa State University, Ames, IA 50014, USA
[4]Genome Informatics Facility, Iowa State University, Ames, IA 50014, USA
[*]e-mail: mash@iastate.edu

Supplementary Information

`pyrpipe` implementation details

Using python classes to *encapsulate* the various "tools" and "data" is the key structural concept of `pyrpipe`. Based on the principle of *abstraction*, `pyrpipe` hides the unnecessary details and provides the user with simple-to-use objects and functions.

Thus, the "tools" can be easily accessed as objects in a re-usable manner, while ensuring that associated data and parameters are consistently accessible within that object and full functionality of the command/tool is retained.

At its core, `pyrpipe` implements the "Runnable" class, which implements all necessary functionality for *importing* and running any executable command/tool via Python. Users may simply create an instance of the "Runnable" class to access any Linux/macOS executable in Python, or can create a new class extending the "Runnable" class with additional specialized functionalities. Users can employ several helper functions defined in two of the `pyrpipe` modules, *pyrpipe_engine*, and *pyrpipe_utils* to access frequently used operations.

Upon creation of a "Runnable" object, the tool options and arguments, if specified in a YAML file, are automatically loaded and stored in the object. These can be dynamically modified during execution. Parameter loading is robust and can ignore incorrect or misspelled options, raising a warning.

The "run" method, implemented in the "Runnable" class, is responsible for executing the commands. The "run" method allows users to specify required dependencies and target files. The required files, if not present, will cause an exception.

The output files are equivalent to "targets" in GNU make; execution is skipped if the output file already exists. Thus, If a workflow is interrupted during execution, it is resumed from the last incomplete step, unless pyrpipe is executed with the *–force* option. Output files for erroneous steps are automatically removed. The "run" method integrates the command and its options and passes them to the *pyrpipe_engine* module, where the commands are executed and extensively logged.

`pyrpipe` allow users to *dry run* the pipeline. During a *dry run*, all the dependencies and targets are checked and any missing dependencies or existing targets are reported to the user. No commands are executed via the *pyrpipe_engine*. Users can examine the *dry run* output to verify the commands and options.

Module name	Class name	API for	Purpose
sra	SRA	sra-tools(v. 2.10.9)[1]	Access NCBI-SRA database and manage Fastq files
mapping	Hisat2<br>Star<br>Bowtie2	Hisat2(v. 2.2.1)[2]<br>STAR(v. 2.7.7a)[3]<br>Bowtie2(v. 2.3.5.1)[4]	Read alignment<br>Read alignment<br>Read alignment
assembly	Stringtie<br>Cufflinks	StringTie(v. 2.1.4)[5]<br>Cufflinks(v. 2.2.1)[6]	Transcript assembly<br>Transcript assembly
quant	Kallisto<br>Salmon	Kallisto(v. 0.46.2)[7]<br>Salmon(v. 0.14.1)[8]	Transcript quantification<br>Transcript quantification
qc	Trimgalore<br>BBDuk	Trim Galore(v. 0.6.0)[9]<br>BBDuk(v. 38.76)[10]	Quality control<br>Quality control
tools	Samtools	Samtools(v. 1.9)[11]	Processing read alignments

Supplementary Table 1. Currently implemented *pyrpipe* modules. Each module contain multiple classes containing APIs for different RNA-Seq tools.

Key	Description
cmd	shell command executed
starttime	Time at the start of execution
runtime	Total runtime
exitcode	The return code
stdout	stdout returned by the program
stderr	stderr returned by the program
objectid	Id of an object used with the command
commandname	Name of the command
python	Python version
os	Operating system
cpu	CPU information
syspath	Python's sys.path
sysmodules	Python's sys.modules
name	Name of the program executed
version	Version of the program
path	Path to the program on disk

Supplementary Table 2. Table showing description of the JSON *keys* stored in `pyrpipe` logs

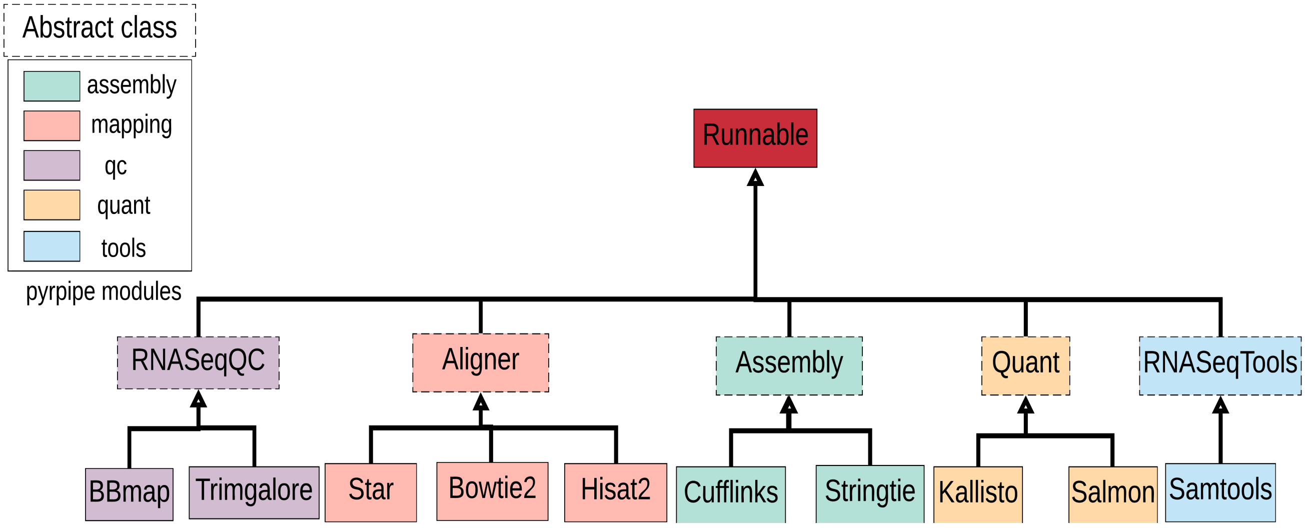

Supplementary Figure 1. A class diagram showing `pyrpipe'` s class hierarchy. Classes in the same `pyrpipe` module share the same color; dotted rectangles represent abstract classes.

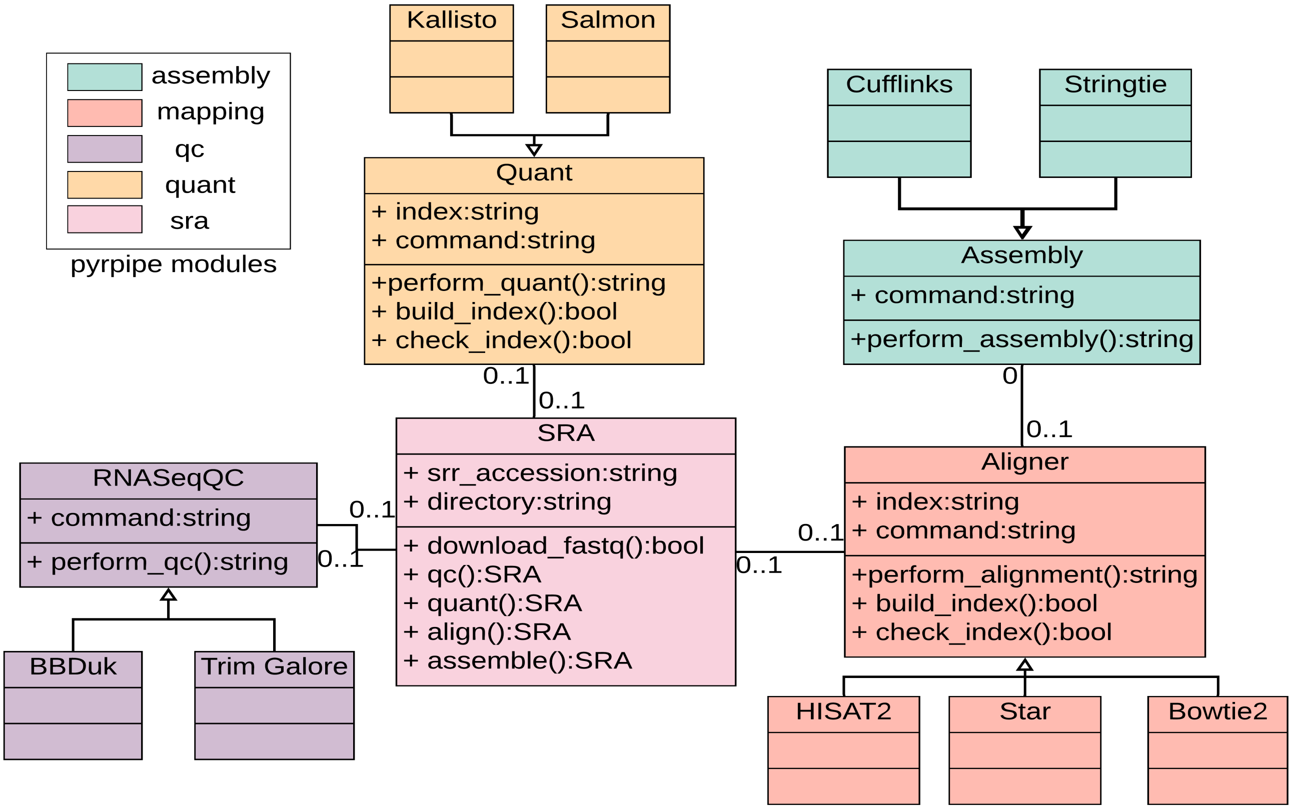

Supplementary Figure 2. A UML class diagram showing `pyrpipe'` s RNA-Seq API classes and relationships among them. Classes in the same `pyrpipe` module share the same color. The numbers, e.g. 0..1, represent UML cardinality which shows the association between the entities. For example, a cardinality of 0..1 between SRA and Quant means that an SRA object may be associated with 1 Quant object.

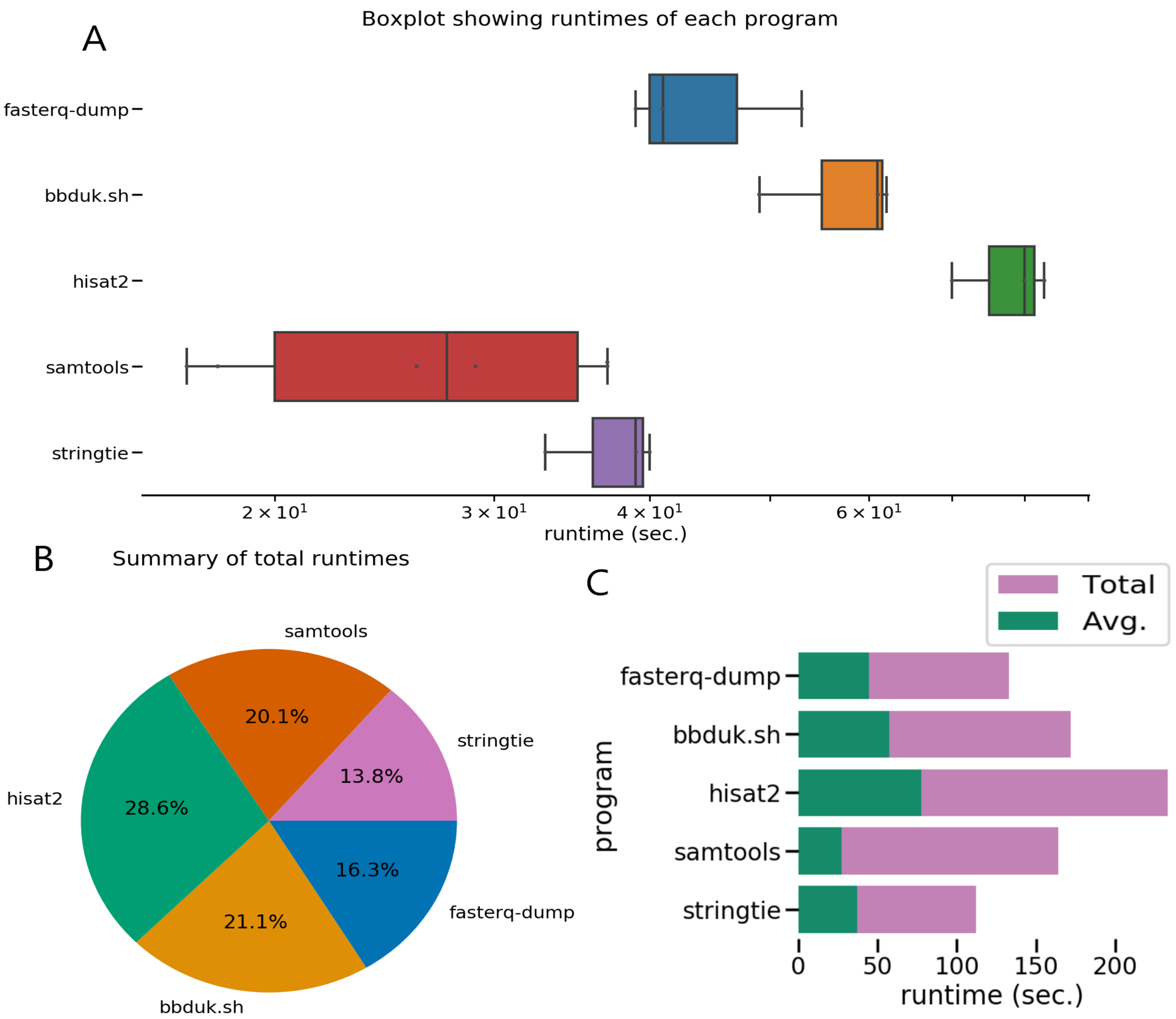

Supplementary Figure 3. Example of plots generated using pyrpipe_diagnostic benchmark command. **A.** Boxplots showing distribution of walltimes of different tools/commands used in the pipeline. **B.** A pie-chart showing how much each tool contributed to total runtime of the pipeline. **C.** Bar plot showing average and total runtimes of each tool in the pipeline. The pipeline code and data is available from https://github.com/urmi-21/pyrpipe/tree/master/case_studies/Athaliana_transcript_assembly.

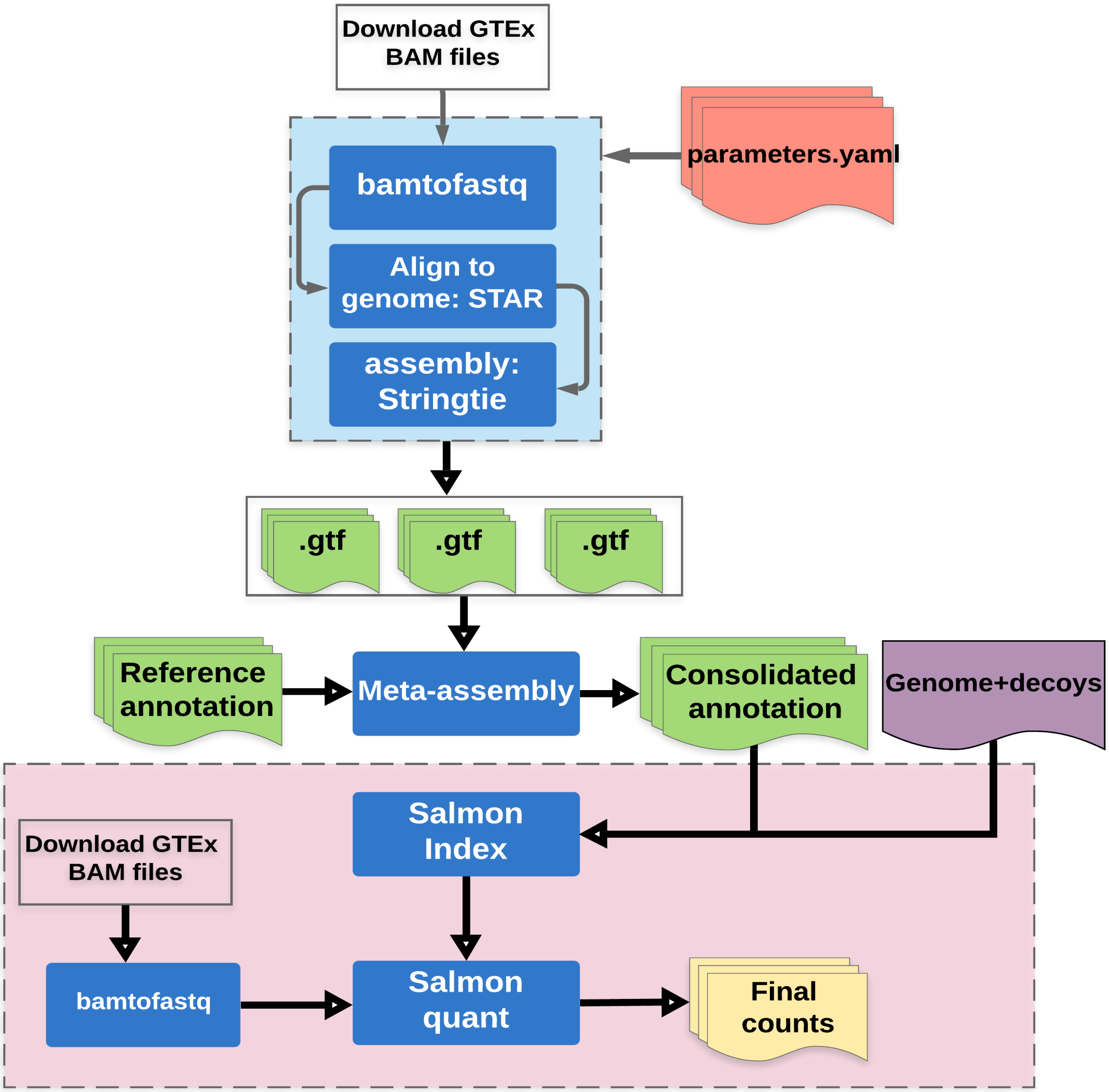

Supplementary Figure 4. A flowchart showing steps used to process GTEx RNA-Seq data. The Alignment and quantification pipelines are implemented in `pyrpipe`. For alignment, BAM files were downloaded from GTEx and converted to fastq using biobambam2[12]. STAR[3] was run in 2-pass alignment mode to align reads to the human reference genome. Stringtie[5] was used to assemble transcripts. Raw data files were deleted as after they were processed to save disk space. Individual transcriptomes were consolidated into single transcriptome using our unpublished meta-assembly pipeline which is similar to[13]. Salmon[8] index was build using human annotated and novel identified transcripts. Human whole genome sequence was used as a decoy. During quantification phase, BAM files were downloaded from GTEx, converted to fastq (using biobambam2) and passed to salmon quant for quantification.

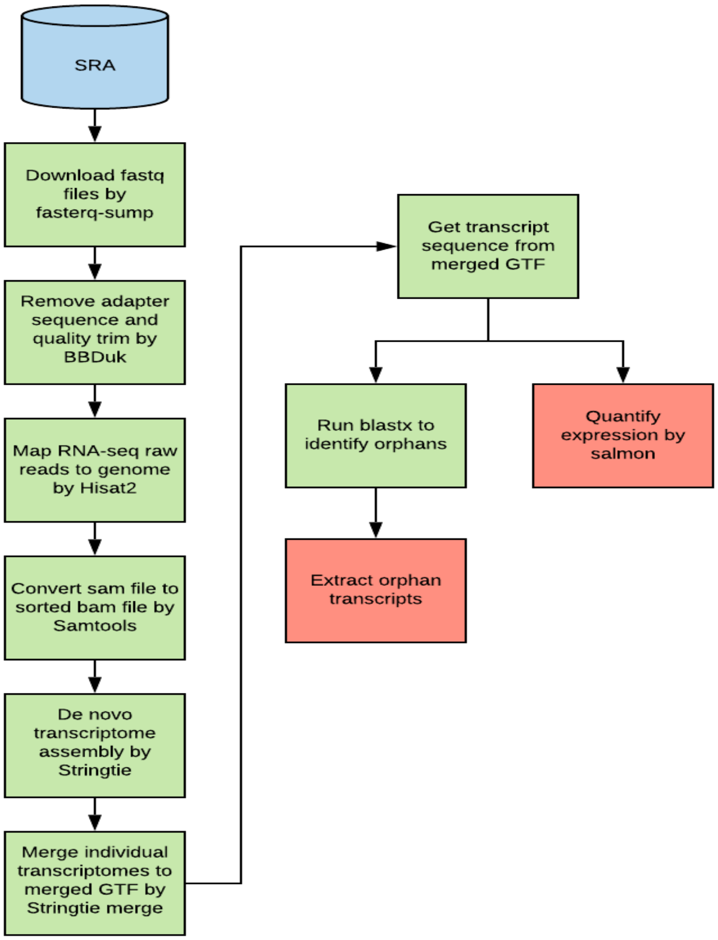

Supplementary Figure 5. A flowchart showing the pipeline implemented in `pyrpipe` to identify potentially orphan coding transcripts in *Zea mays*.

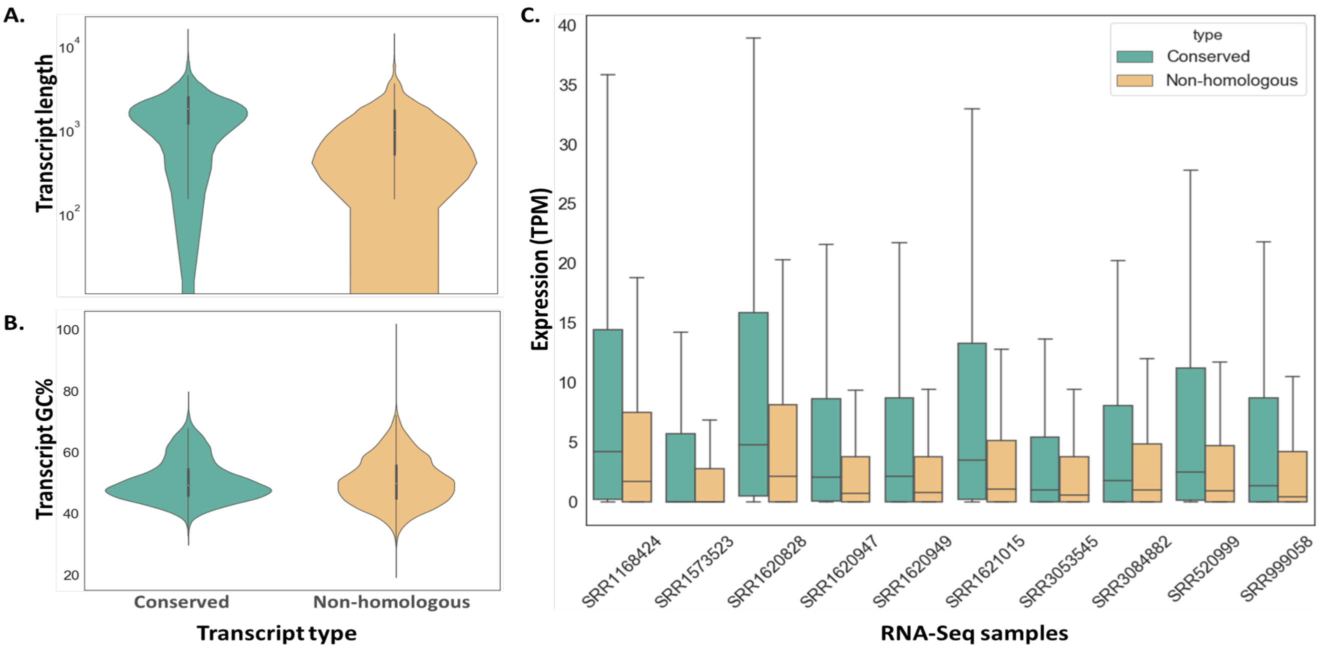

Supplementary Figure 6. Transcriptome assembly from *Zea maize* RNA-Seq data. Ten diverse samples were processed by `pyrpipe`. **A.** length (in nt), **B.** %GC content, and **C.** expression level comparisons of transcripts containing an ORF encoding a protein homologous to an annotated protein of another species (*conserved*) with those without homology to any annotated protein of another species *(non-homologous)*, as identified by our pipeline. The mean length of conserved genes is longer, GC content is similar; the expression level is varied but is generally higher for conserved transcripts. These trends are similar to those for annotated conserved genes vs orphan genes[14] in the genome of the model plant species, *Arabidopsis thaliana*.

```yaml
--runMode: 'alignReads'
--twopassMode: 'Basic'
--outFilterMultimapNmax: '20'
--alignSJoverhangMin: '8'
--alignSJDBoverhangMin: '1'
--outFilterMismatchNmax: '999'
--outFilterMismatchNoverLmax: '0.1'
--alignIntronMin: '20'
--alignIntronMax: '1000000'
--alignMatesGapMax: '1000000'
--outFilterType: 'BySJout'
--outFilterScoreMinOverLread: '0.33'
--outFilterMatchNminOverLread: '0.33'
--limitSjdbInsertNsj: '1200000'
--outSAMstrandField: 'intronMotif'
--outFilterIntronMotifs: 'None'
--alignSoftClipAtReferenceEnds: 'Yes'
--outSAMunmapped: 'Within'
--genomeLoad: 'NoSharedMemory'
--chimSegmentMin: '15'
--chimJunctionOverhangMin: '15'
--chimOutType: 'WithinBAM SoftClip'
--chimMainSegmentMultNmax: '1'
--outSAMattributes: 'NH HI AS nM NM ch'
```


Supplementary Figure 7. An example of YAML (star.yaml) file containing the parameters for the tool STAR[3]. pyrpipe can automatically read tool parameters from YAML files and incorporate them into the pipelines. The parameters specified could be easily modified with-in the python script, if required.

References

1. Sherry, S. & Xiao, C. Ncbi sra toolkit technology for next generation sequence data. In *Plant and Animal Genome XX Conference (January 14-18, 2012). Plant and Animal Genome* (2012).
2. Kim, D., Paggi, J. M., Park, C., Bennett, C. & Salzberg, S. L. Graph-based genome alignment and genotyping with hisat2 and hisat-genotype. *Nat. biotechnology* **37**, 907–915 (2019).
3. Dobin, A. *et al.* Star: ultrafast universal rna-seq aligner. *Bioinformatics* **29**, 15–21 (2013).
4. Langmead, B. & Salzberg, S. L. Fast gapped-read alignment with bowtie 2. *Nat. methods* **9**, 357 (2012).
5. Pertea, M. *et al.* Stringtie enables improved reconstruction of a transcriptome from rna-seq reads. *Nat. biotechnology* **33**, 290 (2015).
6. Trapnell, C. *et al.* Transcript assembly and quantification by rna-seq reveals unannotated transcripts and isoform switching during cell differentiation. *Nat. biotechnology* **28**, 511 (2010).
7. Bray, N. L., Pimentel, H., Melsted, P. & Pachter, L. Near-optimal probabilistic rna-seq quantification. *Nat. biotechnology* **34**, 525 (2016).
8. Patro, R., Duggal, G., Love, M. I., Irizarry, R. A. & Kingsford, C. Salmon provides fast and bias-aware quantification of transcript expression. *Nat. methods* **14**, 417 (2017).
9. Krueger, F. Trim galore. *A wrapper tool around Cutadapt FastQC to consistently apply quality adapter trimming to FastQ files* (2015).
10. Bushnell, B. Bbtools software package. *URL http://sourceforge. net/projects/bbmap* (2014).
11. Li, H. *et al.* The sequence alignment/map format and samtools. *Bioinformatics* **25**, 2078–2079 (2009).
12. Tischler, G. & Leonard, S. biobambam: tools for read pair collation based algorithms on bam files. *Source Code for Biol. Medicine* **9**, 13 (2014).
13. Seetharam, A. S. *et al.* Maximizing prediction of orphan genes in assembled genomes. *BioRxiv* (2019).
14. Arendsee, Z. W., Li, L. & Wurtele, E. S. Coming of age: orphan genes in plants. *Trends plant science* **19**, 698–708 (2014).
